# Herpesvirus Telomerase RNA(vTR)-Dependent Lymphoma Formation Does Not Require Interaction of vTR with Telomerase Reverse Transcriptase (TERT)

**DOI:** 10.1371/journal.ppat.1001073

**Published:** 2010-08-26

**Authors:** Benedikt B. Kaufer, Sascha Trapp, Keith W. Jarosinski, Nikolaus Osterrieder

**Affiliations:** 1 Department of Microbiology and Immunology, College of Veterinary Medicine, Cornell University, Ithaca, New York, United States of America; 2 Institut für Virologie, Freie Universität Berlin, Berlin, Germany; Emory University, United States of America

## Abstract

Telomerase is a ribonucleoprotein complex involved in the maintenance of telomeres, a protective structure at the distal ends of chromosomes. The enzyme complex contains two main components, telomerase reverse transcriptase (TERT), the catalytic subunit, and telomerase RNA (TR), which serves as a template for the addition of telomeric repeats (TTAGGG)_n_. Marek's disease virus (MDV), an oncogenic herpesvirus inducing fatal lymphoma in chickens, encodes a TR homologue, viral TR (vTR), which significantly contributes to MDV-induced lymphomagenesis. As recent studies have suggested that TRs possess functions independently of telomerase activity, we investigated if the tumor-promoting properties of MDV vTR are dependent on formation of a functional telomerase complex. The P6.1 stem-loop of TR is known to mediate TR-TERT complex formation and we show here that interaction of vTR with TERT and, consequently, telomerase activity was efficiently abrogated by the disruption of the vTR P6.1 stem-loop (P6.1mut). Recombinant MDV carrying the P6.1mut stem-loop mutation were generated and tested for their behavior in the natural host *in vivo*. In contrast to viruses lacking vTR, all animals infected with the P6.1mut viruses developed MDV-induced lymphomas, but onset of tumor formation was significantly delayed. P6.1mut viruses induced enhanced metastasis, indicating functionality of non-complexed vTR in tumor dissemination. We discovered that RPL22, a cellular factor involved in T-cell development and virus-induced transformation, directly interacts with wild-type and mutant vTR and is, consequently, relocalized to the nucleoplasm. Our study provides the first evidence that expression of TR, in this case encoded by a herpesvirus, is pro-oncogenic in the absence of telomerase activity.

## Author Summary

The enzyme complex telomerase, with its two main components telomerase reverse transcriptase and telomerase RNA, plays an important role in telomere maintenance. Perturbation of telomere length regulation can ultimately result in cellular senescence (telomere shortening) and is also observed in tumor cells (increased telomere maintenance). Recent studies suggest telomerase RNAs can function independently of the telomerase complex and promote tumor development independently of telomere maintenance. Here we demonstrate that vTR, a herpesvirus-encoded telomerase RNA, serves two distinct functions in MDV-induced tumor formation. vTR has its first function early after infection, when it is part of the telomerase complex and contributes to the survival of rapidly dividing transformed cells. The second function of vTR is independent of telomerase action and essential for formation of solid lymphomas and metastasis. This latter function is likely a consequence of vTR-mediated gene regulation that is at least in part controlled by its interaction with and relocalization of RPL22, a cellular factor involved in T-cell development and virus-induced transformation. Taken together, our study demonstrates that telomerase RNA encoded by a herpesvirus is directly involved in tumor formation *in vivo* in a fashion that is largely independent of its function within an active telomerase complex.

## Introduction

Telomerase is a multi-component ribonucleoprotein complex. One of its main functions is the maintenance of telomeres, a protective structure at the termini of linear chromosomes. The telomerase complex consists of two essential core components, telomerase reverse transcriptase (TERT) and telomerase RNA (TR), which serves as a template for the catalytically active subunit in the elongation of telomeric repeats (TTAGGG)_n_ at the end of chromosomes [1]. TR contains four structural domains, which are highly conserved regions (CR) in all vertebrates: I) the pseudoknot (core) domain, containing the template sequence (CR1); II) the H/ACA box and III) the conserved region (CR) 7 domain, both of which are essential for TR stability and localization; IV) the CR4-CR5 domain, which is required for efficient TR-TERT complex formation, hence telomerase activity and processivity [2], [3]. An essential structure within the CR4-CR5 domain is the P6.1 stem-loop. Base pairing of the P6.1 stem is completely conserved in all vertebrates. Disruption of the base paring of the P6.1 stem was shown to interfere with proper TR-TERT interaction and resulted in absence of telomerase activity *in vitro* and *in vivo* [3]–[5]. In addition, the P6.1 stem-loop was shown to interact with conserved sequences of the template region CR1, which also plays a critical role in the catalytic activity of the telomerase complex [5].

Telomerase activity is tightly regulated and varies amongst cell types. While it is commonly up-regulated in germ-line, stem and cancer cells, it is absent in most somatic cells [6]. The absence of telomerase activity often leads to progressive telomere shortening, known to initiate cellular senescence and irreversible cell cycle arrest. Several tumor-inducing viruses have evolved strategies to evade and subvert this mechanism of cellular senescence, mainly via the up-regulation of TERT, which was shown to be the limiting factor of telomerase activity in some organisms, such as the human and the chicken [7], [8]. It has been suggested that up-regulation of TERT expression and provision of more active telomerase increases the proliferative potential of persistently infected cells, which in turn might be beneficial to accumulate genetic alterations and transformation after infection [8].

One of the most remarkable viruses with respect to the efficiency of the induction of fatal tumors is Marek's disease virus (MDV), a lymphotropic alphaherpesvirus, that causes Marek's disease (MD) in chickens, characterized by neurological disorders, immune suppression and, primarily, malignant T cell lymphomas [9]. The rapid onset of MD-induced lymphomas, as early as 2 weeks post-infection, and high tumor-induced mortality (90–100% in susceptible animals), suggests a direct involvement of virus-encoded oncogenes in the process. The major MDV oncogene, *meq*, encodes a basic leucine zipper (bZIP) transcription factor (TF) that was shown to interact with Rb, cdk2 and p53, proteins involved in cell-cycle control, and several cellular TFs including c-Jun, c-Fos and c-Myc, an oncogene known to regulate TERT expression [10], [11]. In addition, and as a unique feature, the MDV genome harbors two copies of its own TR subunit, termed viral TR (vTR), that shares 88% sequence identity with chicken TR (chTR), contains all four conserved structural TR domains, and was likely acquired from the chicken genome [2]. The 180-kbp linear, double-stranded DNA genome of MDV consists of a long (L) and short (S) unique region (U_L_ and U_S_) flanked by terminal (TR_L_ or TR_S_) and internal (IR_L_ or IR_S_) inverted repeats. Both vTR copies are located in the repeats flanking the U_L_, TR_L_ and IR_L_. Besides the presence of vTR in the TR_L_ and IR_L_, MDV also contains two sets of tandem repeats in very close proximity to the genomic termini that represent perfect telomeres [12].

vTR is expressed during both lytic and latent MDV infection. It is functionally active and was shown to more efficiently induce telomerase activity *in vitro* when compared to its cellular homologue, chTR [13], [14]. Although dispensable for lytic replication *in vitro* and *in vivo*, vTR is required for efficient MDV-induced tumorigenesis, as MDV mutants lacking both copies of vTR were severely impaired in lymphoma formation and dissemination [14].

Recent reports suggest that both TERT and TR may also have roles in tumorigenesis aside from their role in the maintenance of telomere length in rapidly dividing cells [7], [15], [16]. For example, human TR has been shown to restrain activity of ATR, a factor in the DNA damage response pathway, in a telomerase-independent fashion allowing the survival of cells after cellular stress such as UV radiation [17]. Furthermore, knockdown of TR in human cancer cells induced rapid changes in the global gene expression profiles that were independent of telomere maintenance and DNA damage responses. Induced changes in expression levels included genes involved in cell cycle progression (Cyclin G2 and Cdc27) and adhesion (integrin αV), that may have an effect on MDV pathogenesis and tumorigenesis as well [15]. Similarly, expression of vTR in the chicken fibroblast DF-1 cell line that does not exhibit telomerase activity, induced a 2-fold increase of integrin αV expression, suggesting a telomerase-independent function for vTR [14], [15], [18].

One potential interaction partner of vTR is ribosomal protein L22 (RPL22), previously shown to interact with human TR [19]. Besides associating with ribosomes, RPL22 is also involved in the development of T-cells [20], [21], the target of MDV transformation. Epstein-Barr virus (EBV), a herpesvirus that shares many pathobiological similarities with MDV, encodes two small RNAs, termed EBER-1 and EBER-2, that contribute to tumor formation and are highly abundant in latently infected cells [22]. EBER-1 was shown to interact with RPL22 and the interaction resulted in relocalization of RPL22 from the nucleolus to the nucleoplasm. The interaction of EBER-1with RPL22 is associated with enhanced potential for cellular proliferation [22], [23].

In order to elucidate whether MDV vTR has functions that are independent of telomere maintenance and its presence in the telomerase complex, we mutated the P6.1 stem-loop present in CR4-CR5 of MDV-encoded vTR. The mutation was shown to efficiently abrogate vTR-mediated telomerase activity *in vitro*. *In vivo* studies analyzing MD incidence, tumor development and dissemination confirmed that vTR serves functions that are both dependent and independent of the formation of an active telomerase complex. In addition, we identified RPL22 as an interaction partner of vTR and show that it is relocalized upon vTR expression. To our knowledge, the data presented here provide the first *in vivo* evidence that a TR executes functions important for tumor formation that are independent of telomerase activity and likely depend on the alternate usage of RPL22 in the transformation process.

## Results/Discussion

### vTR P6.1 stem-loop mutation efficiently disrupts telomerase activity

**Figure 1:**
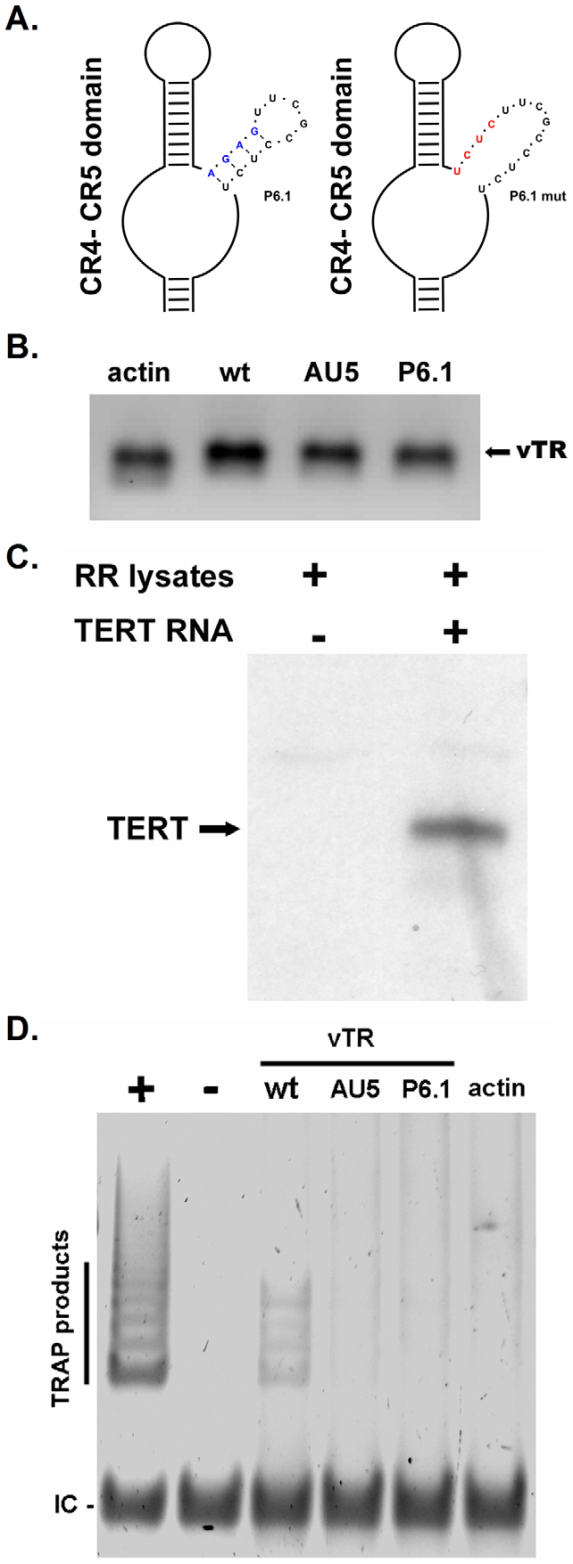
Effect of the P6.1 mutation on telomerase activity. A) Schematic of the CR4–CR5 domain including a detailed representation of the P6.1 stem-loop. Structure of wild-type P6.1 (left) and mutant P6.1 stem-loop (P6.1 mut) (right) is shown. Nucleotide changes of the wt P6.1 stem-loop (blue) are shown in red. B) *In vitro* transcribed β-actin control RNA, wt vTR RNA, vTR containing the P6.1 mutation (P6.1) or a mutation in the template sequence (AU5) was analyzed on a 2% denaturing agarose-formaldehyde gel. Expected vTR size is indicated by the black arrow. C) Chicken TERT-His was translated *in vitro* using rabbit reticulocyte lysates and subsequently analyzed via western blotting using an anti-5x-His antibody. The expected size of TERT-His is indicated with the black arrow. D) Telomerase activity of the *in vitro* transcribed vTR variants was analyzed using gel based TRAP-assays. TRAP products and the internal control (IC) are indicated. The results shown are representative for three independent experiments showing similar results.

To ensure that the disruption of the vTR P6.1 stem-loop, as previously shown for cellular TRs, efficiently abrogates vTR-TERT interaction and consequently telomerase activity, we performed gel-based telomere repeat amplification protocol (TRAP) assays. Base pairing of the P6.1 stem-loop was disrupted by mutating base pairs (bp) 295–298 of vTR from 5′-AGAG-3′ to 5′-UCUC-3′ (Fig. 1A). In order to confirm the absence of telomerase activity via TRAP assay, *in vitro* transcription was used to generate various vTR's (Fig. 1B) that were used in the TRAP assays: wild- type (wt) vTR, vTR containing the P6.1 mutation (Fig. 1A), or, as a negative control, vTR containing a mutation in the template sequence (AU5) resulting in the addition of (TATATA)_n_ repeats that are not amplified in the TRAP assay. Functional chTERT protein was obtained by *in vitro* transcription of a synthetic cDNA followed by translation using a rabbit reticulocyte lysate system (Fig. 1C). In order to reconstitute the telomerase complex, chTERT was incubated with vTR variants or actin control RNA and telomerase activity analyzed by TRAP assays. While TRAP products were readily detected with wt vTR confirming earlier results [13], telomerase activity was undetectable when vTR with the P6.1 mutation was used, as was evident from the absence of TRAP products in reactions containing P6.1mut. Similarly, addition of vTR with a template mutation (AU5) or negative control RNA to the TRAP reaction did not result in telomere elongation (Fig. 1D). Although clearly detectable, few TRAP products were obtained with the vTR-TERT combination. The relatively low activity of reconstituted vTR-TERT compared with the positive control TR could be due to the low TERT levels generated by *in vitro* transcription/translation, the lack of accessory telomerase factors, or a high protein content of the reticulocyte lysates known to reduce TRAP product generation [24].Our results clearly demonstrated, however, that the introduced mutation within the vTR P6.1 stem-loop completely abrogates the formation of an active telomerase complex.

### Construction of MDV bacterial artificial chromosome (BAC) mutants

**Figure 2:**
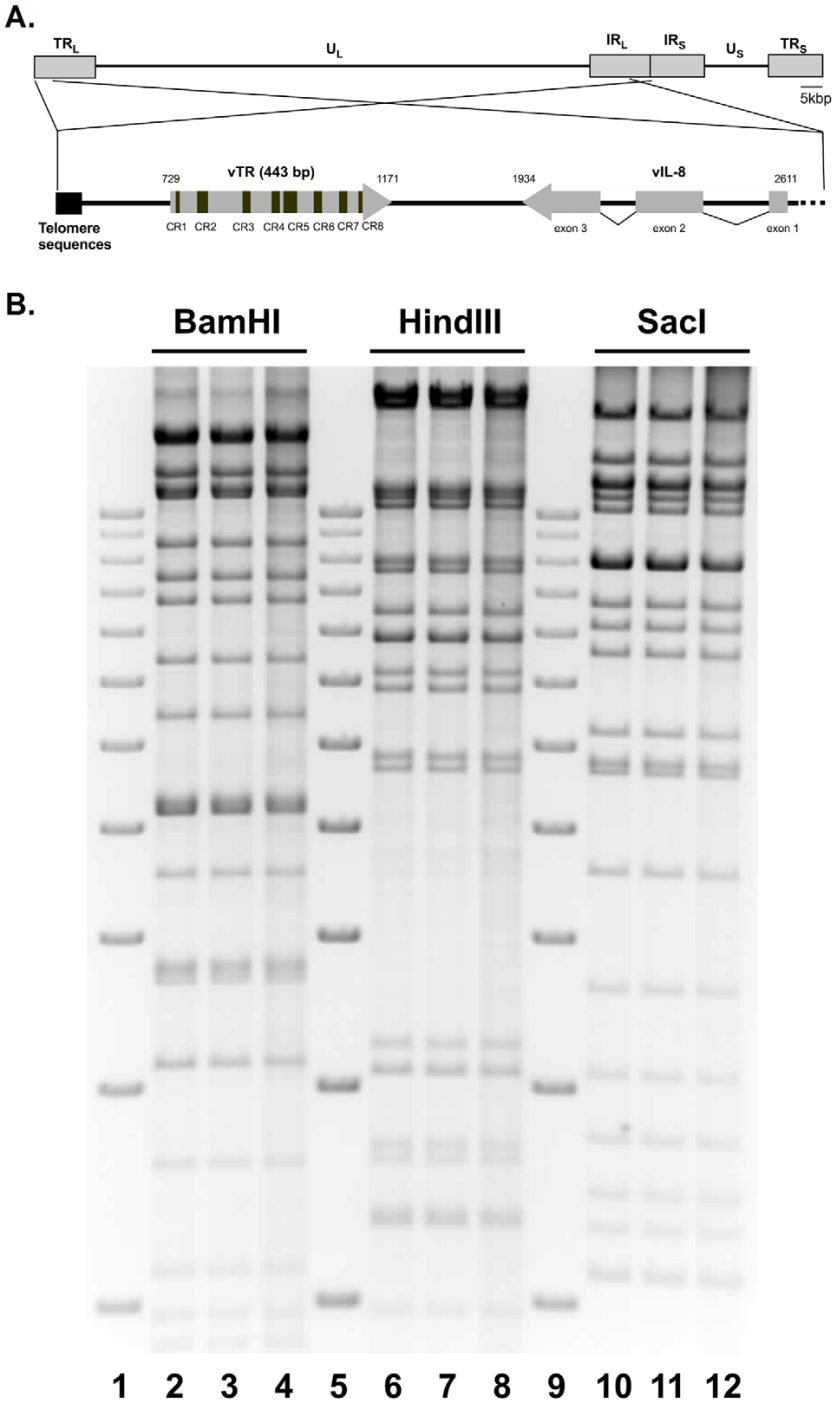
MDV genome organization and P6.1 stem-loop mutation. A) Schematic representation of the MDV genome including the unique-short and -long regions (U_S_, U_L_) flanked by terminal and internal repeat regions (TR_S_, TR_L_, IR_S_, IR_L_). The focus on the vTR containing regions shows the telomeric repeat region present in the MDV genome (left), vTR including its conserved regions (CR) 1–8, and the three exons of the neighboring vIL-8 gene (right). B) Restriction fragment length polymorphism analyses of pRB-1B (lane 2, 6, 10), vP6.1mut (lane 3, 7, 11) and vP6.1rev (lane 4, 8, 12) using the indicated restriction enzymes. Lane 1, 5, and 9 show the 1 kb plus ladder (Invitrogen) ranging from 2 kbp (lowest band) till 12 kbp (highest band) in exact 1 kbp increments.

To determine whether the established tumor-promoting function of vTR is dependent on the formation of an enzymatically active telomerase complex, we manipulated the P6.1 stem-loop in pRB-1B, an infectious BAC clone of the highly oncogenic RB-1B MDV strain (Fig. 2A) [25]. Base pairing of the P6.1 stem-loop was disrupted by mutating base pairs (bp) 295–298 of vTR, as described above, via two-step Red-mediated mutagenesis [26] (Fig. 1A). Two rounds of identical mutagenesis allowed the desired alteration of both copies of the diploid vTR gene within the MDV genome, and the resulting mutant infectious clone was termed pP6.1mut. In addition, a revertant BAC clone (pP6.1rev) was generated in which the original sequence was restored in both alleles. All clones were confirmed by PCR, DNA sequencing and multiple restriction fragment length polymorphism analyses (RFLP) to ensure the integrity of the genome (Fig. 2B). In order to confirm that the mutation did not revert during any of the experimental procedures, DNA of stock viruses used for infection of the animals and viral DNA obtained from tumor cells were analyzed by nucleotide sequencing, which demonstrated that the vTR mutants were genetically stable throughout the experiments.

### vTR-TERT interaction and telomerase activity are dispensable for efficient lytic viral replication *in vitro* and *in vivo*

**Figure 3:**
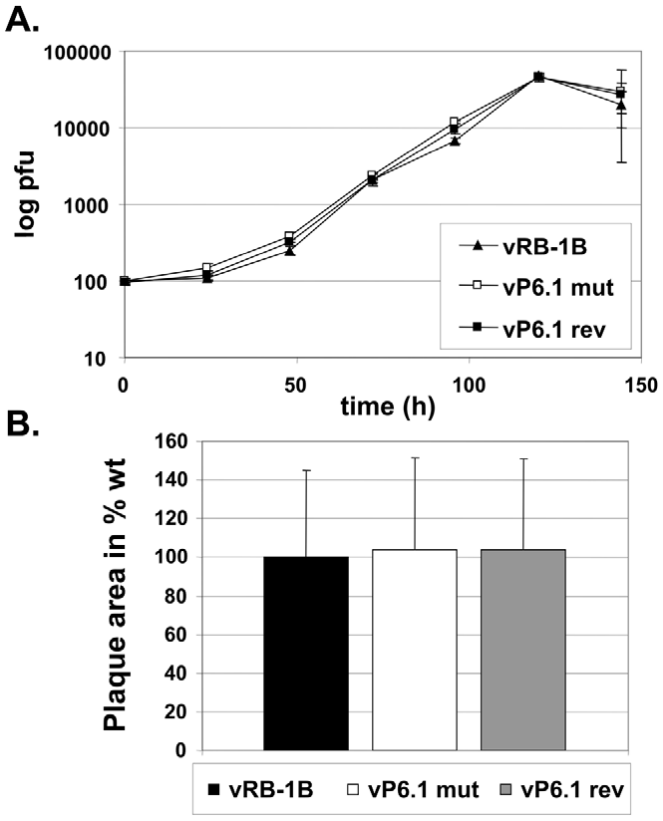
Growth properties of viruses containing the P6.1 stem-loop mutation. A) Multi-step growth kinetics of wt vRB-1B, vP6.1mut and vP6.1rev were performed in triplicates and are shown as means with standard deviations (error bars). B) Plaque size assay. Results are shown for the three recombinant viruses as the relative mean plaque area in percent of 100 randomly selected plaques induced by each of the viruses with the corresponding standard deviations (error bars).

In order to investigate the effect of the P6.1 stem-loop mutation on virus replication *in vitro*, wt pRB-1B, pP6.1mut and pP6.1rev BACs were transfected into chicken embryo cells (CEC) resulting in the reconstitution of recombinant viruses termed vRB-1B, vP6.1mut and vP6.1rev. Multi-step growth kinetics revealed that replication of vP6.1mut was unaffected *in vitro* when compared to that of wt vRB-1B or vP6.1rev (Fig. 3A). In addition, mutation of the P6.1 stem-loop had no effect on the plaque sizes induced by the vP6.1 virus mutant (Fig. 3B). These findings were consistent with previous data on vTR deficient viruses, which had shown that vTR is dispensable for lytic virus growth *in vitro* [14].

**Figure 4:**
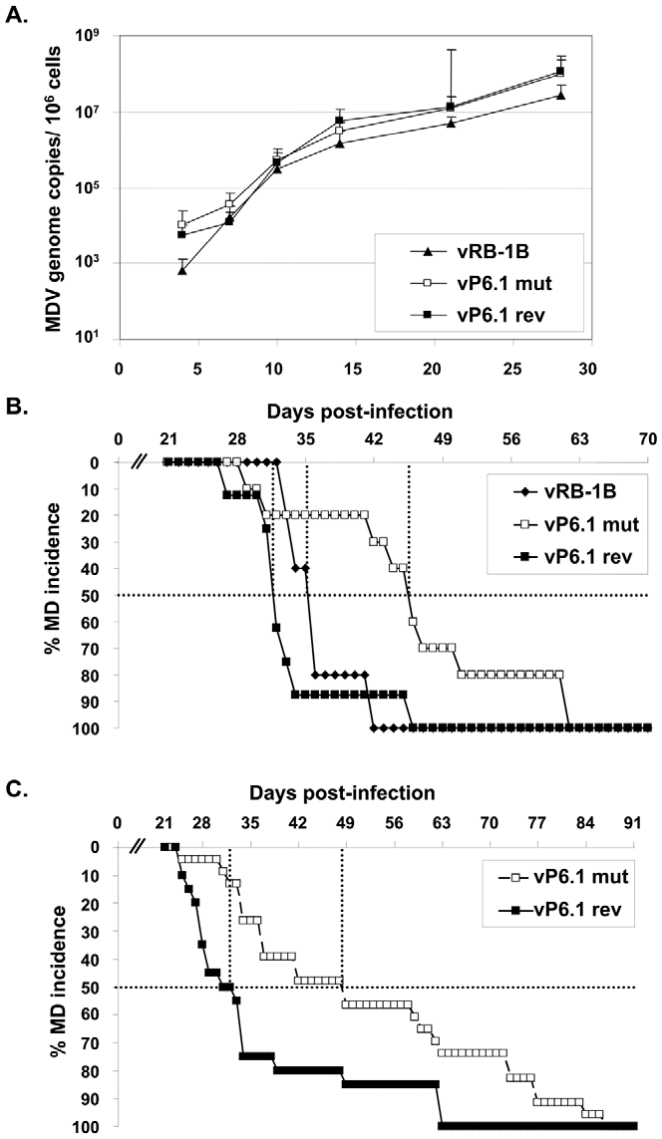
P6.1 stem-loop mutation does not affect lytic replication *in vivo*, but delays MD incidence. A) qPCR analysis of the viral *ICP4* gene and the host *iNOS* gene. Blood samples were taken at 4, 7, 10, 14, 21, and 28 dpi and total DNA was extracted. Mean MDV genome copies/10^6^ blood cells of eight infected chickens per group as determined by qPCR analysis are shown with standard deviations (error bars). B) 1^st^ animal experiment: MD incidence in percent in chickens infected with vRB-1B (n = 5), vP6.1mut (n = 10) and vP6.1rev (n = 8) during the indicated time period C) 2^nd^ animal experiment: MD incidence in percent of vP6.1mut (n = 22) and vP6.1rev (n = 20) during the indicated time period. The time to develop MD in 50% of the inoculated animals (MD_50_) is indicated (dashed line) and was significantly increased in the P6.1mut group (p = 0.0012).

Since efficient lytic replication *in vivo* is considered a prerequisite for efficient lymphomagenesis, we analyzed the replicative potential of the various mutant viruses in the natural host. We infected 1-day-old chickens and monitored virus levels by qPCR using DNA isolated from whole blood obtained by wing vein puncture until 28 days post infection (dpi). MDV is present in peripheral blood mononuclear cells (PBMC) and qPCR analyses showed that vP6.1mut replicated in those cells to levels that were comparable to those of wt vRB-1B or vP6.1rev (Fig. 4A). The results were again consistent with published data on the lytic replication of vTR deficient viruses, which were shown to be fully capable of robust lytic replication [14]. The observed dispensability for lytic replication of vTR-TERT interaction and vTR-mediated telomerase activity in general can be explained by the fact that the initial virus production in chicken B and T cells does not require long-term survival of the host cell or host cell proliferation. Survival of the latently infected host cell is, however, a prerequisite for, or consequence of transformation and tumor formation in general. From the results of the experiments on lytic replication of the P6.1mut viruses we concluded that viruses containing the P6.1 stem-loop mutation are capable of efficient replication in cultured cells *in vitro* as well as in the target cells *in vivo*. Therefore, vTR-TERT interaction mediated by the P6.1 stem-loop and, therefore, telomerase activity is dispensable for MDV replication in vivo.

### Onset of MDV-induced lymphoma is delayed in the absence of vTR-TERT interaction and vTR-mediated telomerase activity

We have previously shown that MD lymphoma formation was significantly reduced in the absence of vTR [14]. To address whether the observed reduction is dependent on the interaction of vTR with TERT, we performed two independent animal experiments in which we monitored the temporal occurrence of virus-induced lymphoma in chickens infected with vRB-1B, vP6.1mut or P6.1rev. In a first animal experiment, we established that abrogation of vTR-mediated telomerase activity markedly delayed the onset of MD lymphomas (Fig. 4B). We observed that the time until development of tumors and occurrence of MD in 50% of the infected animals (MD_50_) was increased from 36 dpi in vRB-1B-infected chickens (n = 5) and 32 dpi in vP6.1rev-infected animals (n = 8) up to 46 dpi in vP6.1mut-infected birds (n = 10).

In a second animal experiment, the clinician examining inoculated chickens was blinded to eliminate subjectivity. In agreement with the results of the first animal experiment, MD_50_ was significantly delayed in vP6.1mut infected chickens (49 dpi, n = 22) when compared to vP6.1rev expressing wt vTR (32 dpi, n = 20) (p = 0.0012). We hypothesize that the observed delay in the development of lymphomas is caused by curtailing telomerase activity mediated by vTR and, consequently, the absence of enhanced telomere maintenance. Such enhanced telomere maintenance, which was shown in MDV-infected animals [27] and is thought to play an important role for the survival of rapidly dividing MDV-transformed cells early in the transformation process, is probably mediated mainly by an interaction between vTR and cellular chTERT. In the absence of the P6.1 stem-loop, the interaction can no longer occur, and, therefore, the pool of transformed cancer stem cells surviving the initial crisis may be reduced.

It was notable, however, that, in contrast to viruses lacking vTR [14], all animals infected with v6.1mut succumbed to MD before termination of the experiment, indicating that vTR has functions independent of the formation of an active telomerase complex. From the results of the animal experiments we conclude that the rapid onset of MD observed in chickens infected with wt MDV (vRB-1B) or the vP6.1rev virus is dependent on telomerase activity that involves vTR-chTERT interaction. Lymphoma formation, however, and fatal disease outcome are efficient even in the absence of enzymatically active telomerase.

### vTR-TERT interaction is not required for efficient tumor dissemination

**Figure 5:**
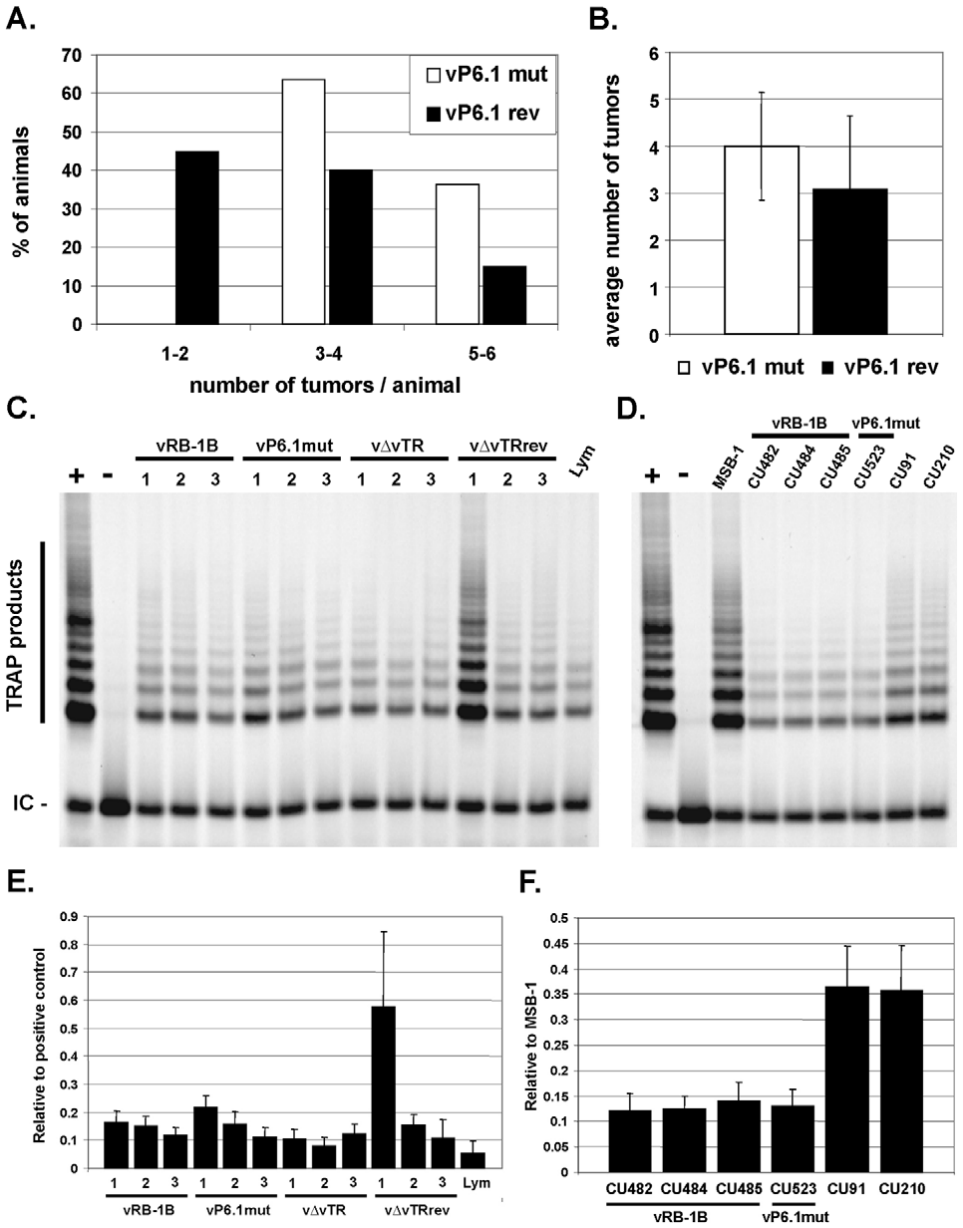
vTR-TERT interaction is not required for efficient tumor dissemination or for telomerase activity in tumor cells. A) Dissemination pattern of vP6.1mut (22 chickens) and vP6.1rev (20 chickens) for *in vivo* experiment 2. Moribund chickens were euthanized, necropsied and evaluated for lymphoma dissemination. Results are shown as percentage of animals with 1–2, 3–4 or 5–6 organs containing lymphomatous lesions. B) Mean number of tumors per animal with standard deviations. The mean number was significantly increased in the P6.1mut group indicated by the asterisk (p = 0.0381). C–D) Telomerase activity in primary tumor cells (C) and clonal LCLs (D) derived from vRB-1B or vP6.1mut infected animals as indicated using the Cy5 gel based TRAP-assay. TRAP products and the internal control (IC) are indicated. E–F) Quantification of telomerase activity in primary tumor cells (E) and established LCLs (F). The data are shown as mean telomerase activity relative to the positive control in three (E) or two (F) independent experiments.

MDV-induced tumor formation and metastasis were previously shown be significantly reduced in the absence of vTR [14]. In addition, our earlier findings of integrin αV up-regulation mediated by vTR alone suggested that malignant lymphoma dissemination may be a result of the action of vTR that is independent of vTR-TERT interaction [14]. To address whether animals infected with the P6.1mut virus, where vTR-TERT complex formation is absent and, hence, more non-complexed vTR is available, would corroborate these earlier findings. We enumerated the gross lesions in infected birds during necropsies on animals that had succumbed to infection. Consistent with our earlier results and the hypothesis that lymphomagenesis and metastasis could be largely determined by vTR action alone, disruption of the P6.1 stem-loop led to a significant increase in the number of solid lymphomas in chickens infected with the vP6.1mut virus when compared to vP6.1rev-infected chickens (p = 0.0016). All vP6.1mut-infected animals developed gross tumors in at least three organs (Fig. 5A). Furthermore, the average number of organs with solid lymphomas was mildly albeit significantly increased from 3.1 in vP6.1rev to 4.0 in vP6.1mut (p = 0.0381; Fig. 5B). We concluded, therefore, that efficient tumor dissemination observed in vP6.1mut-infected animals supported the results of a previous study suggesting that vTR is involved in increased lymphoma dissemination and metastasis [14].

### vTR does not contribute to telomerase activity in MDV transformed tumor cells

In order to address whether telomerase activity was affected in tumor cells derived from vP6.1mut-infected animals, we performed quantitative CY5 gel-based TRAP assays as described by Herbert and coworkers [28]. The experiment showed that telomerase activity was not affected in primary tumor cells derived from vP6.1mut-infected animals when compared to tumor cells recovered from animals infected with parental vRB-1B virus, suggesting that endogenous TR in transformed T-cells can compensate for the telomerase activity mediated by vTR. In addition, we analyzed established, clonal LCLs derived from animals infected with vRB-1B or virus containing the P6.1 stem-loop mutation. vP6.1mut-derived cell lines exhibited telomerase activity comparable to those transformed with wild-type vRB-1B. To address whether vTR contributes to telomerase activity during MDV transformation *in vitro*, we performed TRAP assays using CU91, a retrovirus-transformed T-cell line obtained from chickens with the same genetic background (B^19^B^19^) as the animals from which cell lines after infection with vRB-1B or vP6.1mut were derived. Similarly, the CU210 cell line was used, which was generated by superinfection of CU91 with MDV strain RB-1B [33]. Latent MDV infection in CU210 and, hence, vTR expression, did not increase telomerase activity when compared to the parental CU91 cell line, which showed higher telomerase levels when compared to the MDV-derived cell lines. The high telomerase activity of MSB-1, an MDV-transformed and highly passaged LCL, suggested that such serial passage might select for increased telomerase activity that likely contributes to a profound transformation phenotype that is reflected by very robust proliferation observed for MSB-1 [29]. Taken together, our data suggested that vTR does not contribute to telomerase activity in MDV-transformed tumor cells, which further lends support to the hypothesis of telomerase-independent functions of vTR in the development and dissemination of lymphoma.

### vTR and vTR P6.1 efficiently interact with RPL22

As previously reported, EBV transformation mediated by EBER-1 is dependent on its interaction with RPL22 [22]. In order to determine if wild-type and/or mutant P6.1 vTR interact with RPL22, we performed biotin-RNA pull-down assays. vTR, vTR P6.1, chTR and EBER-1 were found to precipitate RPL22, while biotin-labeled β-actin control RNA did not (Fig. 5C). EBER-1 showed the strongest interaction that was 5.1-fold stronger than that determined for vTR (Fig. 5D), potentially because it contains three independent RPL22 binding sites [30]. The interaction of chTR with RPL22 was reduced by 2.0-fold, indicating that cellular TR does not interact as strongly as that encoded by MDV. vTR P6.1 showed a 1.9-fold increase in precipitated RPL22 when compared to wild-type vTR. This apparently enhanced interaction could be caused by a conformational change of the P6 stem-loop structure, that exhibits high similarity to the EBER-1 stem-loop 3 that is known to interact with RPL22 [30]. In addition, the abrogation of vTR-TERT interaction in MDV infected cells could increase the amount of free vTR available for RPL22 interaction, which may provide an explanation for the increased number of solid tumors found in vP6.1mut infected animals.

### vTR expression affects RPL22 localization

**Figure 6:**
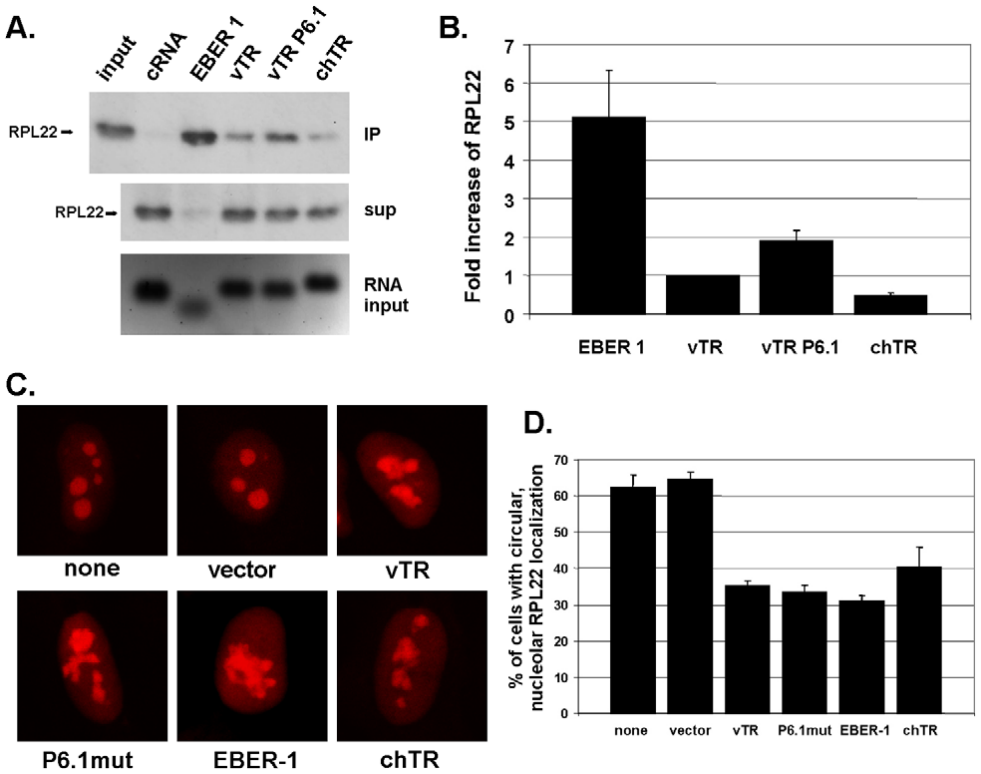
vTR interacts with and affects localization of RPL22. A) Biotin-RNA pull-down assay. Precipitated RPL22 (upper panel), unbound supernatant (middle panel), and RNA input control (lower panel) of indicated RNAs are shown. The figure is a representative of three independent experiments yielding identical results. B) Quantification of three independent Biotin-RNA pull-down assays. Mean RPL22 quantities are shown relative to vTR with standard deviations (error bars). C) RPL22 localization in HeLa cells transfected with pcDNA-RPL22-mRFP and empty vector (pCMS-EFGP), vTR (pCMS-vTR), vTR P6.1mut (pCMS-vTR-P6.1mut), EBER-1 (pSG5-EBER-1) or chTR (pcDNA-chTR). Representative images of cells from three independent experiments are shown. D) Quantification of nucleolar RPL22 localization in HeLa cells. At least 160 individual cells were evaluated for each sample. Data are shown as mean percentages of circular RPL22 localization of three independent experiments with standard deviations (error bars).

Finally, to address whether vTR expression has an affect on the localization of RPL22, we determined RPL22 localization by confocal microscopy in vTR-transfected cells. HeLa cells were co-transfected with an expression plasmid encoding RPL22 that is C-terminally tagged with mRFP (RPL22-mRFP) and plasmids expressing EBER-1, vTR, P6.1mut or chTR. In cells transfected with RPL22-mRFP alone or together with empty vector, RPL22-mRFP localized almost exclusively to the nucleolus as described previously [31]. As shown in previous reports, EBER-1 expression induced relocalization of RPL22; however, RPL22 was not completely absent from the nucleoli, which could possibly be attributed to lower amounts of EBER-1 plasmid DNA used here when compared to earlier reports (Fig. 6C). A relocalization of RPL22 quite similar to that following EBER-1 expression was also detected for vTR, P6.1mut and chTR, suggesting that over-expression of viral as well as cellular TR affects RPL22 subcellular distribution (Fig. 6C–E). Cells with a nucleolar localization of RPL22 were quantified to confirm that the relocalization is a general and not isolated event. As in cells transfected with EBER-1, the number of cells with nucleolar RPL22 localization was clearly reduced after co-expression of vTR, P6.1mut or chTR. Under the conditions used here, efficiency of relocalization of RPL22 was comparable between EBER-1 and the vTR and chTR constructs (Fig. 6), which may suggest that EBER-1 and vTR serve similar purposes in the process of transformation of human and chicken lymphocytes.

### Conclusions

**Figure 7:**
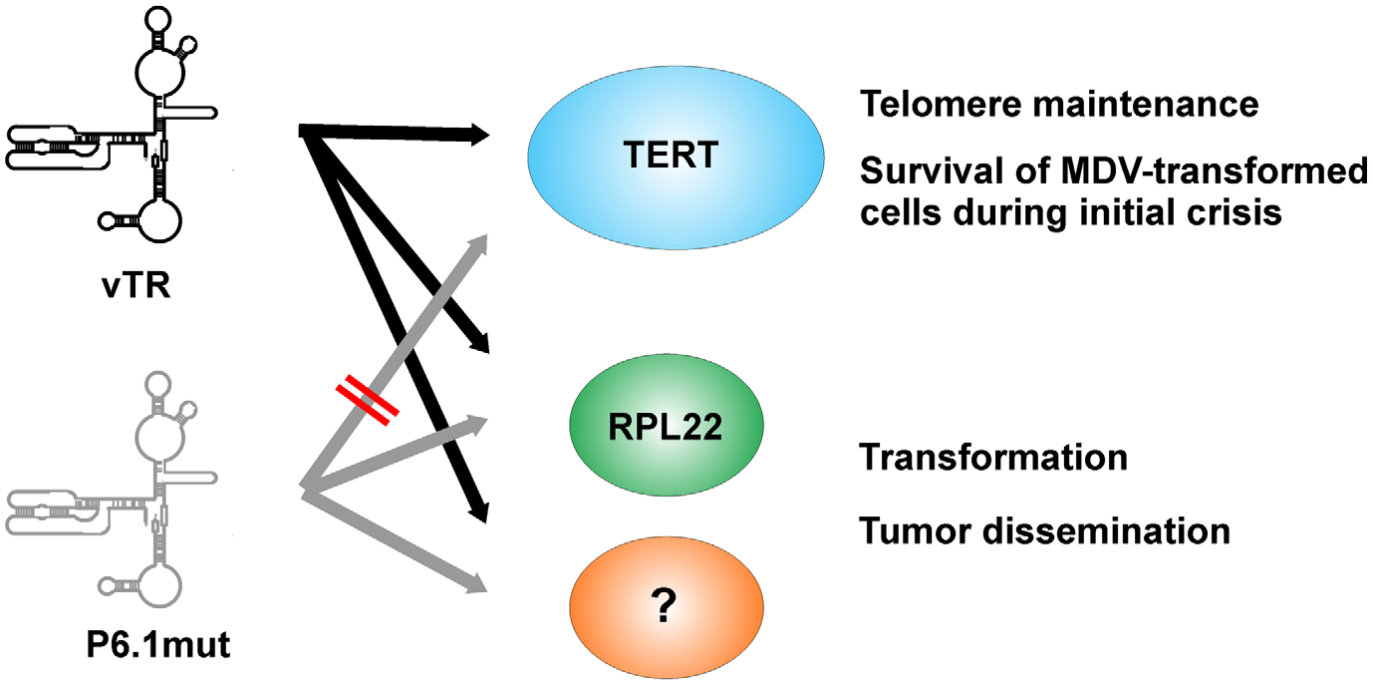
Model of chTR and vTR during MDV infection with vP6.1mut. chTR (gray) and wt vTR are able to interact with TERT (blue) and mediate telomerase activity, which is crucial for the survival of early MDV-transformed cells during initial crisis. P6.1mut vTR (Rose) is not able to interact with TERT and can, therefore, not contribute to telomerase activity. P6.1mut, as well as wt vTR, is able to interact with RPL22 (Red) and potentially also other factors (green) which mainly contributes to transformation and tumor dissemination.

In this report, we demonstrate that the herpesvirus telomerase RNA, vTR, has at least two functions in virus-induced lymphomagenesis. One of its functions is dependent on vTR-TERT interaction, while the other is independent of the formation of an active telomerase complex. The rapid onset of lymphoma formation seems dependent on vTR-mediated telomerase activity because a delay in the development of tumors was observed when vTR-TERT interaction was abrogated. The documented increase in telomerase activity mediated by the presence of vTR in complex with TERT when compared to the presence of cellular TR likely plays an important role in the initial establishment and maintenance of MDV-transformed cells. It may, therefore, facilitate the development of lymphomas by increasing the pool of candidate tumor stem cells (Fig. 7). Functions of vTR that are independent of telomerase activity, however, are needed later in the process and influence homing of tumor cells to various organs, seeding and metastasis. These processes are likely a consequence of TR-mediated gene regulation [15] and the interaction of vTR with RPL22 suggests an alternative mechanism involved in transformation that may be similar to that demonstrated for EBV EBER-1 (Fig. 7). In conclusion, our study demonstrates that TR is directly involved in tumor formation *in vivo*, in a fashion that is independent of its function as an integral component of an active telomerase complex.

## Materials and Methods

### Ethics statement

All animal work was conducted at Cornell University according to national regulations. The animal care facilities and programs of Cornell University meet the requirements of the law (89–544, 91–579, 94–276) and NIH regulations on laboratory animals, and are in compliance with the Animal Welfare Act, PL 279. The College of Veterinary Medicine at Cornell University is accredited by the Association for Assessment and Accreditation of Laboratory Animal Care. All experimental procedures were in compliance with approval of Cornell University's Institutional Animal Care and Use Committee (IACUC, internal approval number: 2002-0085).

### Cells and viruses

MDV transformed lymphoblastoid T cell lines (LCL) were generated as described previously and cultivated in RPMI medium 1640 plus 10% FBS and 8% chicken serum at 41°C in a humidified atmosphere of 5% CO2 [32], [33]. The MSB-1 cell line was kindly provided by Mark S. Parcells (University of Delaware, Newark, DE) whereas the CU91 and CU210 [34] cell lines were kindly provided by Karel A. Schat (Cornell University, Ithaca, NY). CECs were prepared from specific-pathogen-free embryos and maintained as described previously [35]. Recombinant viruses were reconstituted in CECs by CaPO_4_ transfection of purified BAC DNA as described previously [36], [37]. The lox-P-flanked mini-F sequences within the infectious clones were removed by cotransfection with a Cre recombinase expression vector (pCAGGS-NLS/Cre) [36]. Removal of the mini-F sequences was ensured by analyzing recombinant virus stocks by analytic PCR as described previously [36]. Virus propagation as well as determination of virus growth kinetics and plaque sizes were performed as described previously [38].

### Generation of mutant MDV

pP6.1mut and pP6.1rev were generated by two-step Red-mediated recombination [26], [36]. Primers used for the mutagenesis are given in Table 1.

**Table 1:** Primers used for cloning and mutagenesis.

Construct name		sequence (5′ → 3′)
pP6.1mut	for	CGCAGGCCGCGGTCGGCCGGCACCCGCCATTGCCGCCGCGATCTCTTCGCCTCTGTCAGCCTCGTAGGGATAACAGGGTAATCGATTT
	rev	GCCGCATCTCCCGGGCGCCGCCGAGGCTGACAGAGGCGAAGAGATCGCGGCGGCAATGGCGGGGCCAGTGTTACAACCAATTAACC
pP6.1rev	for	GCAGGCCGCGGTCGGCCGGCACCCGCCATTGCCGCCGCGAAGAGTTCGCCTCTGTCAGCCTCGTAGGGATAACAGGGTAATCGATTT
	rev	CCGCATCTCCCGGGCGCCGCCGAGGCTGACAGAGGCGAACTCTTCGCGGCGGCAATGGCGGGGCCAGTGTTACAACCAATTAACC
pUC119-vTR	for	CATGCCTGCAGTAATACGACTCACTATAGGGACACGTGGCGGGTGGAAGG
	rev	GATCCTCTAGATGCGCATGTGGGAGCGACGCC
pCMS-vTR	for	CTCGAGAATTCTGCAGATCCTCGGACACGTGGCGGGTGGAAG
	rev	CTAGTGGATCCCCCGGGCTGCAGGCGTGTGGGAGCGACGC
pUC119-vTR-P6.1 and pCMS-vTR-P6.1	for	**TC** TTCGCCTCTGTCAGCCTCG
	rev	**GA** TCGCGGCGGCAATGGC
puc119-vTR-AU5	for	**TATAA** CGGAGGTATTGATGGTACTGTC
	rev	**TATATA** ACACAGCGGAGCCTTCCAC
pcDNA-chTERT-His	for	ACGCGTGGCGGGTGGAAG
	rev	GCGTGTGGGAGCGACGCC
pcDNA-chTR	for	ACGCGTGGCGGGTGGAAG
	rev	GCGTGTGGGAGCGACGCC
pcDNA-RPL22	for	GCCGCCATGGCGCCCGT
	rev	GTCCTCCTCCTCCTCCTCCTCC
pcDNA-RPL22-mRFP	for	TAGATCTCGAGTATGGCCTCCTCCGAGGAC
	rev	TCAGTCTCGAGTTACAAGGCGCCGGTGG

Underlined sequences indicate restriction enzyme sites. Bold indicates mutated sequences.

### *In vivo* experiments

SPF P2a (MHC: *B^19^B^19^*) chickens were inoculated intra-abdominally with 500 to 2,000 plaque-forming units at day 1 of age and housed in isolation units. All experimental procedures were conducted in compliance with approved Institutional Animal Care and Use Committee (IACUC) protocols (internal approval number: 2002-0085). Chickens were evaluated for symptoms of MDV-induced disease on a daily basis and examined for gross tumors when clinical symptoms were evident.

### DNA extraction and qPCR assays

DNA was extracted from whole blood and MDV genomic copies were determined by qPCR [39], [40]. Briefly, MDV DNA copy numbers were detected using primers and probe specific for the *ICP4* locus and normalization was achieved using chicken inducible nitric oxide synthase (*iNOS*) genome copies.

### Cloning of vTR variants, chTERT, chTR and RPL22

vTR was amplified from pRB-1B and subsequently cloned into the *PstI* and *XbaI* sites of the pUC119 plasmid resulting in plasmid pUC119-vTR. The T7 promoter was inserted at the 5′ end of vTR via a 5′ overhang in the vTR-T7-for primer. vTR was also cloned into the *EcoRI* and *BamHI* sites of the pCMS-EGFP plasmid resulting in plasmid and pCMS-vTR. Mutation of the template (AU5) and the P6.1 stem-loop was done based on pUC119-vTR and pCMS-vTR by Phusion Site-Directed Mutagenesis (Finnzymes Inc.) according to the supplier's instructions and resulting in pUC119-vTR-AU5, pUC119-vTR-P6.1 and pCMS-vTR-P6.1 respectively. Chicken TERT (chTERT) was obtained as a synthetic, codon-optimized sequence from GenScript (Piscataway, NJ USA), PCR amplified including an upstream Kozak sequence and inserted into pcDNA3.1/V5-His TOPO (Invitrogen) containing a 5′ T7 promoter, resulting in plasmid pcDNA-chTERT-His. Chicken TR (chTR) and RPL22 was amplified from chicken DNA and inserted into pcDNA3.1/V5-His TOPO, resulting in pcDNA-chTR and pcDNA-RPL22-His. For the expression of fluorescently labeled RPL22, we amplified mRFP from pmRFP-1 [41] and inserted it into the *Xho*I site of pcDNA-RPL22-His resulting in pcDNA-RPL22-mRFP. Oligonucleotides used for amplification are given in Table 1.

### *In vitro* transcription

vTR variants, chTR, EBER-1, or β-actin were transcribed using the Maxiscript T7 kit (Ambion) following the manufacturer's instructions where the linearized plasmids pUC119-vTR, pUC119-vTR-AU5, pUC119-vTR-P6.1, cDNA-chTR, pSG5-EBER-1 (a kind gift of Dr. Rona Scott, Louisiana State University Health Science Center, Shreveport, LA) and pTRI-β-actin (Ambion) served as templates. Biotin-labeled RNAs were generated using the biotin RNA labeling mix (Roche). chTERT and RPL22 were transcribed via the mMESSAGE mMACHINE T7 Kit (Ambion) according to the supplier's recommendation using linearized pcDNA-chTERT-His or pcDNA-RPL22 as templates. RNAs were purified via the RNeasy Kit (Qiagen), analyzed on a 2% denaturing agarose-formaldehyde gel, and quantified with a NanoDrop 1000 (Thermo Scientific).

### *In vitro* translation of chTERT

*In vitro* transcribed chTERT-His or RPL22-His RNA was used for *in vitro* translation using the Rabbit Reticulocyte Lysate System (Promega) according to the manufacturer's protocol. chTERT-His and RPL22-His expression was analyzed by western blotting, using a mouse anti-5xHis antibody (Qiagen).

### TRAP assay

*In vitro*-transcribed vTR variants (1 µg) were incubated with 1 µL of *in vitro* translated chTERT for 1 h at 30°C to reconstitute the telomerase complex. Telomerase activity was subsequently determined using the *TRAPeze* gel-based telomerase detection kit S7700 (Chemicon) following the manufacturer's instructions or the CY5 gel-based TRAP assay as described by Herbert and coworkers [28].

### Biotin-RNA pull-down assay

4 µL in vitro translated RPL22-His was mixed with 3 nmol biotin labeled vTR, vTR P6.1, chTR, EBER-1 or β-actin control RNA and incubated in binding buffer (150 mM NaCl, 50 mM Tris pH 7.0, 0.1% Tween20, 1 µg of tRNA, 0.5 mM DTT, 0.5 mM PMSF) containing 10 µg tRNA for 1 h at 37°C. 20 uL EZview Strepavidin beads (Sigma) were washed with binding buffer and added to the setup. After binding occurred for 1 h at RT, supernatant was collected and beads washed 7 times with binding buffer containing 1 µg tRNA. Precipitated and unbound protein was analyzed by western blotting, using a mouse anti-5xHis antibody (Qiagen).

### RLP22 localization

5×10^4^ HeLa cells seeded on coverslips in 24-wells were transfected using Lipofectamin 2000 (Invitrogen) with 200 ng pcDNA-RPL22-mRed and 500 ng of either empty vector (pCMS-EGFP), pCMS-vTR, pCMS-vTR P6.1, pCDNA-chTR or pcDNA-RPL22. At 24 h after transfection, cells were examined using an SP5 confocal microscope system (Leica). Images were taken and RPL22 localization evaluated in at least 160 individual cells per sample.

### Statistical analysis

Significant differences in MD incidence were determined using the Wilcoxon rank-sum test (Fig. 4C). Significant differences in tumor distribution were determined using Chi-Square test (Fig. 5A). Significant differences in mean tumor incidences were determined using Student's *t* test (Fig. 5B).
